# Sacral Neuromodulation: Device Improvement and Current Applications in Urology

**DOI:** 10.3390/medicina60030509

**Published:** 2024-03-20

**Authors:** Marco Spilotros, Salvatore Gerbasi, Francesco Lasorsa, Gaetano de Rienzo, Lorenzo Balducci, Pasquale Ditonno, Giuseppe Lucarelli

**Affiliations:** 1 Andrology and Kidney Transplantation Unit, Department of Precision and Regenerative Medicine and Ionian Area-Urology, University of Bari “Aldo Moro”, 70124 Bari, Italy; marco.spilotros@uniba.it (M.S.);; 2 Radiology Unit, University of Bari “Aldo Moro”, 70124 Bari, Italy

**Keywords:** sacral neuromodulation, overactive bladder, bladder pain syndrome, neurogenic lower urinary tract dysfunction

## Abstract

Sacral neuromodulation (SNM) offers a therapeutic approach to urological patients suffering from idiopathic overactive bladder (OAB) syndrome, with or without incontinence and non-obstructive urinary retention (NOR), who are not responding to or are not compliant with conservative or medical therapies. The exact mechanism of action of SNM is not fully understood but modulation of the spinal cord reflexes and brain networks by peripheral afferents is regarded as the main pathway. Over the years, surgical techniques improved, leading to the development of the modern two-stage implantation technique. The quadripolar lead is positioned percutaneously under fluoroscopy guidance through the third sacral foramen following the trajectory of S3. The procedure can be performed under local or general anesthesia with the patient in prone position. Current applications of sacral neuromodulation in urology are increasing thanks to the recent improvements of the devices that make this a valuable option not only in conditions such as overactive bladder and non-obstructing urinary retention but also neurogenic lower urinary tract dysfunction.

## 1. Introduction

Magendie in the early 19th century investigated electric stimulation of the spinal nerve roots in young dogs to achieve muscle contraction, showing how transection of the dorsal segment caused lack of sensation while leaving unaltered the motor function [1]. Following this preliminary evidence, Saxtorph, in 1878 [2], directly stimulated the bladder in patients with urinary retention via a metal transurethral catheter, while McGuire, performing direct bladder stimulation on dogs, demonstrated that multiple pairs of electrodes could uniformly increase bladder pressure [3]. These findings represented the basis for the development of new electrodes and wires until Caldewell in 1965 first reported his experience with pelvic floor muscle stimulation and its correlation with urinary incontinence [4,5]. Further improvements in this field were due to Brindley who, in the early 1970s, began experimentation with sacral root stimulation and its application in paraplegic patients with urinary incontinence, reporting his experience with the implantation of sacral anterior root stimulators [6]. These preliminary findings contributed to outlining the role of the pudendal nerve in bladder capacity modulation and the correlation between sphincteric contraction and the inhibition of detrusor activity. Sacral root neuromodulation was then introduced in this context as a modulator of external sphincter function and detrusor activity, creating the fundamentals for the current concept and technologies of sacral neuromodulation. Sacral neuromodulation (SNM) offers a therapeutic approach for urological patients suffering from idiopathic overactive bladder (OAB) syndrome, with or without incontinence and non-obstructive urinary retention (NOR), who are not responding to or are not compliant with conservative or medical therapies. Encouraging results have been demonstrated also in bladder pain syndrome (IC/BPS) and neurogenic lower urinary tract dysfunction (NLUTD), but further evidence is necessary in these fields. The exact mechanism of action of SNM is not fully understood but modulation of the spinal cord reflexes and brain networks by peripheral afferents is regarded as the main pathway. Two main theories exist about the sacral neuromodulation mechanism of action. According to the first one, SNM creates afferent pulses capable of overwhelming or interfering with the impaired neural activity at the base of overactive bladder syndrome [7]. The second theory affirms that SNM produces a modulation of neural circuits, regulating pathways in both the peripheral and central nervous system. Axonics^®^ and Medtronic, respectively, with their Axonics r-SNM (rechargeable sacral neuromodulation) and InterStim™ systems, are the main manufacturers of sacral neuromodulation devices.

Sacral neuromodulation represents a well-established treatment and in 25 years more than 375,000 patients have been treated worldwide with the Interstim^TM^ system, which currently represents the most popular device implanted for SNM [8].

## 2. Description and Evolution of the Lead

The development of SNM began in the 1980s. Tanagho and Schmidt in 1981 observed how continuous stimulation of the sacral root S3 could modulate detrusor and sphincter activity [9]. The first implantable sacral nerve stimulator was developed in the early 1990s and approved by the US Food and Drug Administration (FDA) for the treatment of urge incontinence (1997) and urgency/frequency and non-obstructive urinary retention (1999). Over the years, surgical techniques improved, leading to the development of the modern two-stage implantation technique with a tined lead, described by Spinelli et al., that offered the possibility to perform this procedure in a minimally invasive manner, under local anesthesia, and avoiding deep incisions and additional fascial anchoring [10]. Since then, significant developments have been applied to sacral neuromodulation devices; one breakthrough was the development of rechargeable systems, which avoid patients having to frequently undergo battery replacement, and another was magnetic resonance imaging (MRI)-safe devices. Interstim II™, Interstim Micro™, Interstim X™ and Axonics^®^ therapy are the sacral neuromodulation systems marketed by Axonics^®^ (Axonics^®^, Inc., Irvine, CA, USA) and Medtronic (Medtronic, Minneapolis, MN, USA) and have been implanted in more than 300,000 patients worldwide since their introduction [11]. Newer devices have a significantly reduced size, which facilitates implantation and increases patient tolerance (Table 1) [8,12]. 

The main differences between these systems are their size and rechargeability. Interstim II™ is a 44 mm × 51 mm implant not requiring recharging; it needs to be replaced after a variable period of time that can reach 5 years according to the voltage of the program used. Interstim Micro™ is the smallest available device (17 mm × 47 mm) with a volume reduction of about 80% when compared to the standard InterStim™ II and an approximately 49% reduction compared to the Axonics device; it requires charging for 20 min a week and needs to be replaced every 15 years [4]. Axonics r-SNM is a 23 mm × 45 mm device requiring charging for one hour every month and it needs to be replaced every 15 years. Rechargeability represents a significant improvement in sacral neuromodulation for two reasons: a rechargeable battery means a smaller pulse generator, as clearly demonstrated by the Interstim Micro™ volume, and it requires less battery changes over time. It is estimated, as mentioned above, that the “life span” of a rechargeable system can reach 15 years, compared to a maximum of 5–7 years for a non-rechargeable device [13,14]. All these devices are MRI-compatible. Full-body MRI compatibility has significantly increased the fields of application of sacral neuromodulation, considering that a large number of patients with sacral neuromodulation systems require MRIs, and in the pre-MRI-compatibility era that was a reason for device explantation in up to 23% of the cases [15,16]. The need for periodic MRIs in neurologic patients represented a relative contraindication for neuromodulation; the spread of MRI-compatible systems has now extended these therapeutic options to this population [17].

## 3. Surgical Aspects

**Figure 1:**
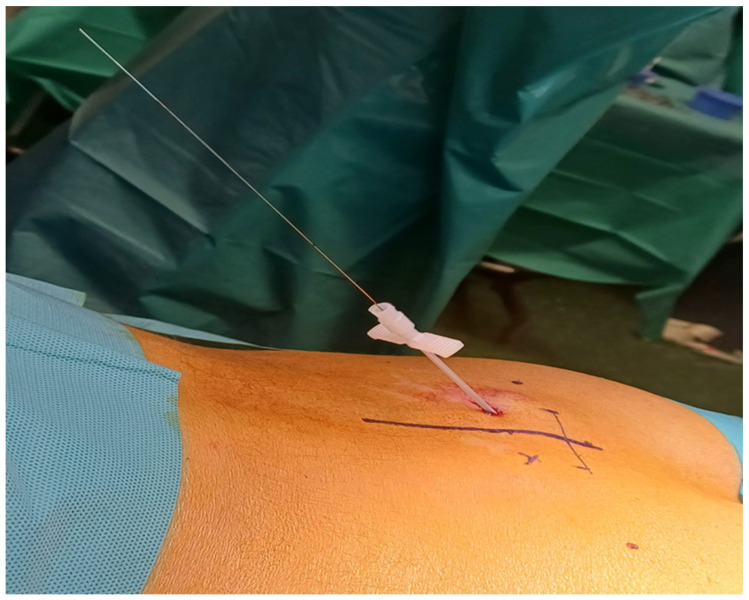
The 60° entry point of the needle during the testing phase is marked 2 cm above a line connecting both ischial spines with patient in prone position and 2 cm lateral to the midline (M.S. personal collection).

Sacral neuromodulation is performed in two stages. During the first stage, the permanent lead is implanted and connected to an external stimulator, while, in the case of therapeutic response in the trial period, in stage II the lead is connected to a subcutaneous implantable pulse generator (IPG). Preoperative intravenous antibiotics are given before the procedure and aseptic techniques are implemented during the procedure. At present, a standardized antibiotic prophylaxis for sacral neuromodulation does not exist but strict attention should be given particularly to the length of treatment, considering the possible related risks [18]. The French Association of Urology and the Neuro-Urology Committee suggested alternative prophylactic regimens including intravenous cefotetan/cefoxitin 2 g, amoxicillin-clavulanic acid 2 g or, in the case of allergy, vancomycin 15 mg/kg or clindamycin 600 mg (grade B recommendation). A recent survey of high-volume sacral neuromodulation providers showed that all implanters administered antibiotics preoperatively, most commonly cefazolin or vancomycin, and 81% recommended 5 to 7 days of antibiotic treatment postoperatively, most commonly cephalexin and trimethoprim-sulfamethoxazole, while only 19% did not prescribe postoperative antibiotics [19,20]. The quadripolar lead is positioned percutaneously under fluoroscopy guidance through the third sacral foramen following the trajectory of S3. This is crucial to increase the number of therapeutic programming options for UUI and NOR, and to reduce the voltage to achieve an adequate response and consequently prolong battery life [21]. Proper lead positioning has demonstrated a reduction in SNM revisions from 32% to 3% [22,23]. The procedure can be performed under local or general anesthesia with the patient in prone position. In the case of general anesthesia, long-acting muscle relaxants are contraindicated to avoid inhibition of the motor response to electric stimulation. Typical sensory responses to S3 stimulation are perceived by the patient in the anal, perineal and vaginal areas, while perianal contractions and flexion of the ipsilateral big toe are expected as motor responses. Correct lead placement is essential. Technically, the lead deployment in the S3 foramen with outward lateral curvatures following the course of the sacral nerve has been related to a better intraoperative response at low amplitude [24]. Technical advances and in particular the tined lead introduced by Spinelli have made implantation of the lead less prone to migration and consequently have decreased the false negative rate during the trial period [10]. Proper motor and sensory responses for all four contacts at electrode stimulation amplitudes of <2 volts represent a guarantee of success providing an adequate therapeutic stimulation from numerous electrodes and a lower probability of undergoing device revision [25]. Motor response has to be considered as a tool for correct lead placement: a closer position of the lead to the target nerve guarantees a better motor response at a lower amplitude of stimulation; a higher number of electrodes with good responses enhances the possibility to “reprogram” around a defective electrode to another one that gives therapeutic benefit and reduces the need for surgical revision; and a lower amplitude of stimulation requires less frequent battery changes [26]. A lower revision rate was demonstrated also in the case of satisfactory big toe motor response due to the stimulation of a more precise and therapeutically significant target nerve compared to perianal motor response [27]. If the stimulation is regarded as satisfactory by the clinician, the lead is deployed along the S3 root and is connected to an external temporary pulse generator which is left in place during the trial period. Between the trial and the second stage of the procedure, the clinical response of the patient is checked, and in the case of 50% improvement in his symptoms, the pulse generator is implanted. The patient is placed in prone position and the buttocks are held apart to make the perianal region visible during the procedure. The buttock or the lower abdomen are usually the target sites to place the generator in a subcutaneous pocket. During the test procedure, a needle is inserted into the third sacral foramen and the sensitive/motor response is checked, applying current through an external simulator. The location of the S3 foramen is pinpointed approximately 9 cm cephalad of the sacrum slope and 1 to 2 cm lateral to the midline bilaterally. The foramen may be also identified by palpating the cephalad portions of the sciatic notches on each side and drawing a connecting line through the midline of the sacrum; one cm on either side of the midline along the intersection will represent the location of the S3 foramen. The author follows as a radiological landmark a line connecting both ischial spines with the patient in prone position; a point 2 cm above and lateral to the midline is then marked as the entry point of the needle (Figure 1). 

**Figure 2:**
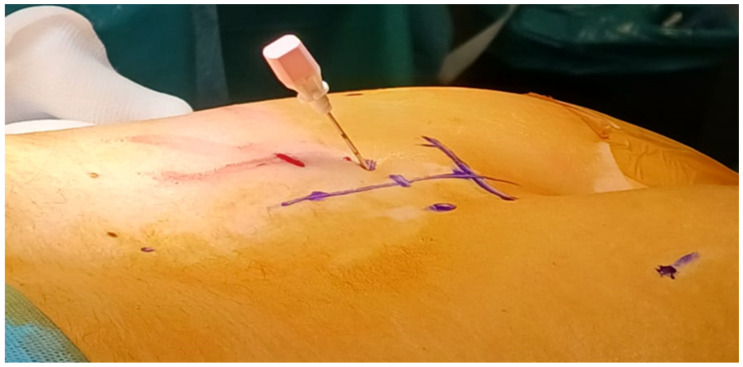
A stylet in inserted into the needle as wire for the correct insertion of a trocar through the S3 foramen (M.S. personal collection).

The needle should be inserted into the sacral foramen at an angle of about 60 degrees. The needle is placed inside the ventral foramen, considering that the S3 root runs alongside the pelvis. In the case of adequate responses, a stylet in inserted into the needle and an introducer sheath is placed (Figure 2). 

**Figure 3:**
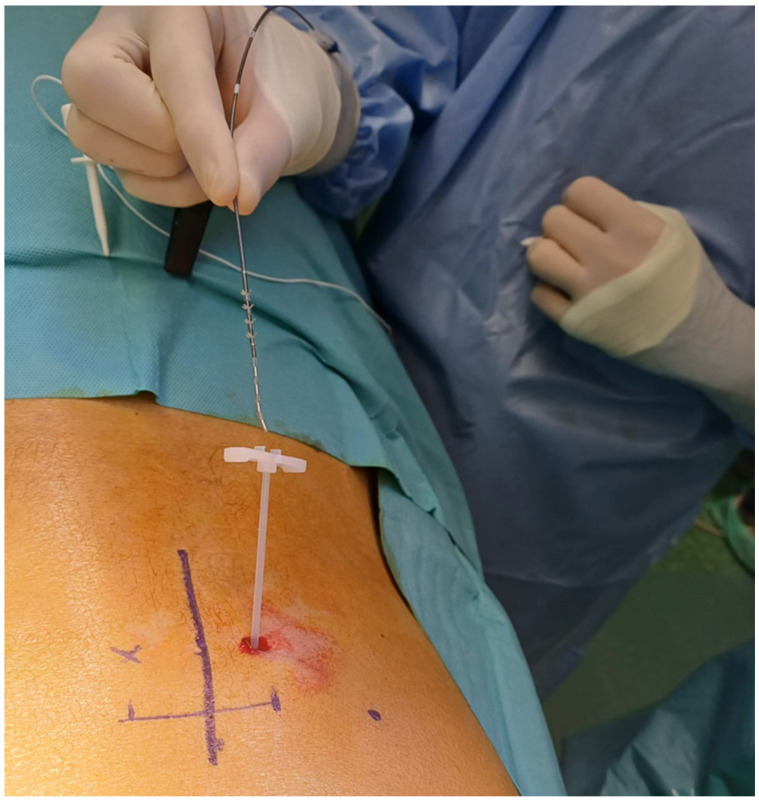
The quadripolar electrode is inserted through a trocar following the route of the S3 root (M.S. personal collection).

Through the introducer sheath, the quadripolar electrode is placed following the route of the S3 root (Figure 3). 

**Figure 4:**
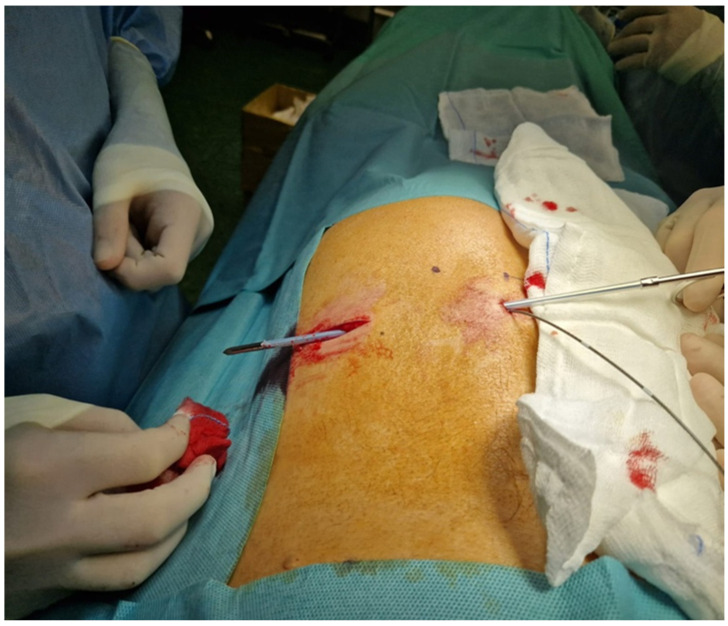
Tunnelization of the electrode to the upper buttock pouch. In the case of a successful trial period in the same pocket, the electrode will be connected to the permanent IPG (M.S. personal collection).

The electrodes are generally positioned such that electrodes 2 and 3 overcome the ventral surface of the sacrum. As mentioned above, in this phase it is recommended to check the response of each electrode, considering that achieving a good sensitive and motor response at low voltage means higher chances of success and a lower revision rate. The lead is tunnelized from the entry point to the upper buttock pouch where the reprogrammable pulse generator will be eventually placed (Figure 4). 

A 3 to 4 cm incision in the upper lateral buttock is made below the beltline and the permanent lead is connected to the percutaneous extension lead wire in a subcutaneous pouch, and this is transposed using a tunnelling device to the contralateral side of the back. During the testing phase, which usually lasts at least 3 days, the electrode is connected to an external simulator and in the case of therapeutic response it will be connected to the IPG. In this second stage, generally performed under local anesthesia, no fluoroscopy is required; the patient is placed in prone position and the stage I buttock incision overlying the lead connection is re-opened, the percutaneous extension wire is removed and the permanent lead is connected to the IPG. It is mandatory to tailor a subcutaneous pocket large enough to avoid tension, erosion of the device and discomfort for the patient.

## 4. Applications and Contraindications

SNM is a treatment option for patients with idiopathic overactive bladder (OAB) syndrome, with or without incontinence and non-obstructive urinary retention (NOR), who have failed to respond to conservative or medical therapies [17]. Its application for bladder pain syndrome and neurogenic lower urinary tract dysfunction (NLUTD) with low risk of upper urinary tract deterioration is still limited. A total of 91% of patients treated with SNM would recommend it to a friend in the same condition, and 84% and 82% reported, respectively, significant improvement in their urological symptoms and stable outcomes at 5 years [28].

Overactive bladder syndrome (OAB) is characterized by urinary urgency, with or without urgency urinary incontinence, usually with increased daytime frequency and nocturia, if there is no proven infection or other obvious pathology [29,30]. Seventeen percent of European adults reported OAB according to a population-based prevalence study. A case-control analysis on 10,000 men and women with and without OAB found that approximately 36% of men and 43% of women over the age of 40 had symptoms of OAB [31]. SNM has been demonstrated to be a reliable and successful approach in treating patients affected by overactive bladder. The AUA and ICS give different levels of recommendation for the use of SNM in OAB. According to the AUA, “Clinicians may offer sacral neuromodulation as third-line treatment in a carefully selected patient population characterized by severe refractory OAB symptoms or patients who are not candidates for second-line therapy and are willing to undergo a surgical procedure. (Evidence Strength Grade C)” [32]. According to the ICS, “SNM can be offered to patients with OAB with or without incontinence who fail to respond to or are intolerant of conservative and medical therapies. (Level of Evidence: I; Grade of Recommendation: A)” [17]. Several studies have demonstrated the effectiveness of SNM in both “dry” and “wet” OAB. 

The effectiveness of SNM in OAB patients has been largely demonstrated. Hassouna MM et al. reported, in 51 patients affected by “dry” OAB over 12 centers and randomized, statistically significant improvements at 6 months in voiding diary results in terms of number of voids daily (16.9 ± 9.7 to 9.3 ± 5.1), volume per void (118 ± 74 to 226 ± 124 mL.) and degree of urgency (rank 2.2 ± 0.6 to 1.6 ± 0.9) [33]. Similar results were described by Siegel et al. more than 20 years ago in a multicenter, prospective study reporting after 3 years post-implantation, showing a 50% reduction in leaking episodes per day in the 59% of patients with UUI, while after 2 years, 56% of the urgency–frequency patients showed a more than 50% reduction in voids per day [34]. In cases of “wet” OAB, positive results at medium and long term have been demonstrated in patients treated with SNM. A trial by Schmidt compared 34 patients with severe “wet” OAB undergoing implantation and 42 patients treated with medical therapy for 6 months and with neuromodulation in cases of the failure of first-line therapy [35]. After 6 months, the immediate implant group showed significant improvements in terms of the number and severity of UI episodes and number of daily pads, compared to the control group. Eighteen months after the treatment, its effectiveness remained consistently high. Siegel et al. investigated changes in the quality of life and safety of sacral neuromodulation 5 years after InterStim™ implantation, demonstrating a success rate of 82% (95% CI 76–88) in patients with urinary urge incontinence (mean reduction from baseline of 2.0 ± 2.2 leaks per day) and subjects with urgency–frequency (mean reduction of 5.4 ± 4.3 voids per day). Improvements were observed in all ICIQ-OABqol measures [28].

In a recent multicenter open-label randomized trial, sacral neuromodulation and intradetrusor injections of 200 U Onabotulinumtoxin A were compared in 381 women with refractory urgency urinary incontinence, showing a small daily improvement of uncertain clinical importance along with a higher risk of urinary tract infections and the need for transient self-catheterizations in the botulinum arm [36]. 

According to the ICS, “SNM is an effective treatment for Fowler’s Syndrome, voiding dysfunction and chronic nonobstructive urinary retention (CNOR) (Level of Evidence: I; Grade of Recommendation: A)” [17]. In patients diagnosed with CNOUR, intermittent self-catheterization (ISC) is usually recommended as the first-line approach, but in those patients who are unable or unwilling to perform ICS, sacral neuromodulation should be taken into consideration as an alternative option. 

A prospective, randomized clinical trial including SNM and a total of 177 patients with NOR showed a clinical success rate of 71% following the implantation at 5 years. After 18 months, 70% of the patients who had received SNM experienced a reduction of more than 50% in volume per catheterization. Additional studies revealed that 69% of the patients who received an implant no longer required catheterization after 6 months of treatment. Moreover, an extra 14% experienced a significant decrease of over 50% in the amount of urine drained through catheters. After 6 months, the implant group had a success rate of 83%, in contrast to a 9% success rate in the control group. Another 5-year follow-up study evidenced an important improvement in terms of mean volume per catheterization and mean number of catheterizations [37]. Jonas et al. demonstrated that the early deactivation of the device results in a significant increase in residual volumes [38]. According to Van Voskuilen et al., the 60% of individuals treated were catheter-free after an average follow-up duration of 15.5 months [9,39]. In patients where it has not been possible to eliminate intermittent catheterization, the most important parameter has been the frequency of catheterizations. Five studies reported a 59.6–77.9% reduction in mean number of catheterizations per day [40]. Another important parameter for evaluating the success of SNM in CNOUR is post-void residual (PVR) volume. Both Denzinger and Mehmood et al. observed significant decreases in mean PVR, respectively, of 93% and 61% [41].

IC/BPS (interstitial cystitis/bladder pain syndrome) is a medical condition characterized by frequent urination, strong urge to urinate, nocturia and, above all, pain in the bladder, urethra and pelvic region. The available evidence supporting the use of SNM in individuals with IC/BPS is low and it is regarded as a fourth line of treatment option. This indication is justified by the presence of small observational case studies demonstrating a success rate for SNM for IC/BPS from 48% to 72% [42]. While SNM might be viewed as a possible fourth-line treatment for IC/BPS, there is limited evidence indicating its effectiveness in treating non-IC/BPS chronic pelvic pain; so, in these patients, SNM should not be suggested as a treatment choice. However, pelvic pain is not a contraindication in patients with concomitant voiding symptoms such as urgency; therefore, pelvic pain does not contraindicate SNM in individuals who also experience concurrent urinary problems like urgency or frequency [43,44]. 

A recent systematic review on the efficacy and safety of sacral neuromodulation (SNM) in patients with non-neurogenic lower urinary tract dysfunction showed an overall dry rate between 43% and 56% with improvement of ≥50% in leakage episodes between 29% and 76%. Less consistent outcomes were observed in interstitial cystitis/bladder pain syndrome patients [45]. Currently, one of the most important controversies about the indication of SNM concerns neurological patients. There is growing evidence, based mostly on case series, about the use of sacral neuromodulation for treating neuro-urological symptoms, but due to the lack of RCTs it remains unclear which neurological patients are most suitable [46]. Nowadays, SNM has been used mostly in patients with stroke, Parkinson’s disease (PD), MS and incomplete spinal cord injury, showing similar efficacy when compared to non-neurogenic populations. PD patients have demonstrated similar rates of trial phase success compared to the general population in retrospective series and, according to Greenberg et al., SNM therapy should be considered among the treatment options for PD patients with overactive bladder symptoms, considering the 60% of patients who experienced ≥50% improvement in urinary parameters [47]. Urodynamic parameters associated with obstruction may be predictive of SNS failure in PD patients and may help guide patient selection. The ICS advises that the use of SNM should be restricted to American Spinal Injury Association (ASIA) D and E in individuals who have an intact sensation of bladder filling and motor function preserved below the neurological level [17]. The probability of success for SNM in patients with upper motor neuron damage could be higher than in those with lower motor neuron injury. This is because upper motor neuron injury tends to maintain the sensitivity and contractility of the detrusor, in contrast to lower motor neuron injury which can impair these functions [48]. According to a recent meta-analysis, the success rates for the test and permanent stages of this treatment are 46% and 76%, respectively [49]. 

With regards to individuals with multiple sclerosis (MS) with overactive bladder (OAB) and detrusor sphincter dyssynergia (DSD), SNM has demonstrated encouraging outcomes [50]. However, for MS patients who are diagnosed with NOR, the success rate for SNM has been reported to be low. A review including small retrospective and prospective series by Puccini et al. reported a subjective cure rate of 45% and patients’ reported satisfaction of 85% with stable results up to 7 years of maximum follow-up [51]. 

Only small series results on sacral neuromodulation in patients with spina bifida are available and further, more robust evidence is needed. A retrospective study on 29 ambulatory spina bifida patients showed a significant improvement in the mean maximum cystometric capacity, compliance and maximum detrusor pressure, especially in those without chronic urinary retention [52]. Chen et al. reported their experience in 33 patients treated with SNM between 2012 and 2021, confirming a low success rate (25.93%) in patients with chronic urinary retention compared to encouraging results in cases of urgency–frequency (63.16%) and urge urinary incontinence (61.11%) [53]. 

In a recent systematic literature review and meta-analysis on SNM in patients with nLUTD (neurogenic detrusor overactivity, non-obstructive urinary retention or a combination of both), the pooled success rates of test stimulation and permanent SNM were 66.2% and 84.2%, respectively, with significant variation according to the neurogenic conditions [54].

## 5. Prediction of Success

Although many studies have been conducted about SNM overall outcomes, nowadays there is a scarcity of literature examining specific clinical features that can predict the effectiveness of SNM devices. In a retrospective chart review, Tara Nikonow Morgan et al. analyzed the results of 198 women, of whom 92.4% completed the stage II implant [11,55]. Women > 65 were found to have a lower probability of completing the second stage of the implant procedure. Additionally, this group had a higher likelihood of needing a revision, although this trend did not reach the threshold of statistical significance. According to Pizarro-Berdichevsky et al. and Richter et al., elderly women have a greater probability of experiencing lead revisions and encounter poorer results [25,56]. Previous treatment with pelvic floor physical therapy (PFPT) is found to have an adverse effect on the success of patients’ outcomes after surgery. Specifically, 50.0% of individuals who did not receive physical therapy (PT) prior to SNM reported successful outcomes after the second stage of implantation, compared to only 22.5% of those who had received PT before. In addition, it has been observed that poorer initial results on standardized questionnaires are linked to a greater likelihood of failure. According to Ranjana Jairam et al., currently the only reliable predictive factor for treatment success in SNM is test stimulation. Even though numerous investigations have assessed predictive elements in individuals with overactive bladder (OAB) or non-obstructive retention (NOR), no consistent variables have been discovered that can be utilized as definitive standards for patient selection in clinical practice. Numerous studies have demonstrated that younger and female patients generally exhibit a higher success rate in SNM. Evidence of detrusor overactivity in patients with OAB at urodynamic study does not seem to have a negative predictive impact according to several studies [57]. Additionally, it was observed that an acontractile detrusor significantly lowers the success rate in patients with NOR. As regards the type of lead, there is only one prospective study with a 2-year follow-up suggesting that a curved stylet can be a positive predictive factor for test success, likely due to the fact that the curved lead aligns with the naturally curving path of the sacral nerve roots, resulting in a more accurate positioning of the lead [58]. According to Jacobs et al., the use of a curved stylet to position the lead resulted in excellent motor responses in all four electrodes with amplitudes below 2 volts [59]. In this perspective, Marinkovic et al. stated that women who exhibited motor responses at lower voltages were more likely to successfully complete stage II implantation [60]. A perioperative motor response is thought to be crucial for successful test stimulation. In particular, an SNM lead positioned next to the S3 nerve root triggers both anal contractions and big toe dorsiflexion when each of the four electrodes on the lead is stimulated. Nevertheless, it is preferable to obtain both motor and sensory responses during the test, because neither of the two alone can accurately predict a successful outcome. 

## 6. Complications

No life-threatening complications have been reported in the literature. Between 15% and 42% experience discomfort at the implant site, making this the most common complication related to this procedure [23,61]. Pain at the ipsilateral site of lead placement (ranging from 5.4% to 19.1%), discomfort in the leg (18%) and the development of an infection (5.7% to 6.1%) are not infrequently reported. Implantable pulse generator (IPG) site discomfort has become less common since the decision to change the preferential site for implanting the neurostimulator from the anterior abdominal wall to the current upper buttock location [62]. Indeed, according to several studies, discomfort is reported to be only 10% for patients implanted at the buttock IPG site, as compared with 15–34% of patients with an anterior abdominal placement [34,63]. Obviously, the development of even smaller devices has allowed the surgeon to make a smaller incision and pocket, with less discomfort for the patient [15,64]. Lead migration happens less frequently when using the tined lead, but it is still possible. Deng et al. conducted a study on 235 patients who were implanted with the tined lead and found that the rate of lead migration was 2.1% [65]. Fortunately, pocket infection is not frequent, but it is a severe complication necessitating the removal of the device [16,66]. Infection after stage I or stage II seems to occur in 2–12% of cases, with an incidence which decreases with adequate antibiotic coverage at the time of implantation, although there is currently no established guideline for antibiotic coverage after SNM [33,37,63,67]. A recent review performed by Clifton et al. states that the only significant risk factor for infection after an IPG (implantable pulse generator) manipulation could be a pre-implant condition of non-obstructive urinary retention [68]. Risk factors have been suggested, such as comorbidities, a prolonged test phase duration, the requirement for surgical reinterventions and pocket hematoma [69].

## 7. Controversial Issues

The role of intermittent stimulation is still a controversial topic. A potential advantage of on-demand stimulation is that it can help keep the nervous system from becoming accustomed or “adjusted” to continuous stimulation. This accommodation may happen when patients with a suboptimal symptom control experience have reduced sensation and keep increasing the intensity of stimulation. In theory, intermittent stimulation may delay or prevent accommodation and ultimately extend the lifespan of the battery. Nowadays, the possibility to implant rechargeable devices exists, thus rendering less useful the intermittent stimulation in this regard. Furthermore, having greater autonomy in controlling the neuromodulator can be an advantage for the patient, as well as being perceived as a burden. In a systematic review from 2020, Roman et al. stated that for patients with urinary tract dysfunction, there were no significant differences in objective outcomes when comparing standard vs. intermittent stimulation [70]. However, there were observed variations between standard and intermittent stimulation regarding subjective assessments, thereby suggesting that short cycling intervals are advantageous when compared to traditional stimulation [71,72]. When extended cycling intervals were compared to continuous stimulation, contrasting results emerged, with Cadish LA stating that there was a decreased quality of life and Hoen suggesting that long cycling intervals led to lower symptom intensity for patients [73].

Unilateral versus bilateral stimulation has been investigated in a few studies with controversial results. Laurent Wagner et al. recruited individuals suffering from primary OAB, excluding those with secondary OAB, or pelvic or neurological conditions [74]. In terms of OAB improvement, between unilateral and bilateral testing before final implantation, the study showed no significant difference between the two groups regarding urinary frequency, number of urge incontinence episodes and number of urinary urgency episodes. Furthermore, there were more complications with bilateral stimulation. In conclusion, according to this study, carrying out a systematic bilateral sacral stimulation before final implantation did not appear to enhance the success rate when compared to the unilateral approach in OAB patients.

In 2011, Tom A T Marcelissen performed a pilot study to assess the efficacy of bilateral stimulation after unilateral therapy failure [75]. Fifteen patients with failure after unilateral implantation were assessed. Three patients were excluded because of doubts about lead migration, and among the remaining 12, only 4 patients had a successful response to percutaneous nerve evaluation. Three of them were implanted with a contralateral lead and after 1 year only 2 had a successful outcome, so only a subset of patients seemed to benefit from the use of bilateral stimulation subsequent to unilateral therapy failure. Wagner, in a recent randomized trial, compared bilateral and unilateral testing in 55 patients with primary overactive bladder and showed no significant difference in terms of improvement in urinary frequency, number of urge incontinence episodes and number of urinary urgency episodes. The same author reported more complications in the bilateral group [74]. Non-superiority of bilateral vs. unilateral testing was confirmed in a further randomized trial [76]. Unilateral vs. bilateral stimulation remains a controversial issue and further investigations are needed to determine the effectiveness and eventually the predictors of success for bilateral stimulation. The safety of sacral neuromodulation during pregnancy is based on series with small numbers of patients. In a systematic review of 2023, Hanieh Salehi-Pourmehr et al. evaluated pregnant women who had undergone SNM previously. The results showed that when the device was turned off, there was a greater proportion of preterm labors (39.1%) compared to the 5.3% recorded when the device was in the on status. In addition, with the device in the on mode, 92.1% of women had full-term pregnancies, while with the device deactivated only 47.8% experienced a full-term pregnancy. The results showed that women who turned off SNM during pregnancy had a higher rate of preterm labor. So, SNM activation in pregnancy seems to be safe and effective. Mahran A. et al. in a systematic review described the results in 26 pregnancies: SNM stayed on in 8 pregnancies and was deactivated in 18 pregnancies. In the latter group, 7 had recurrent urinary tract infections and 2 requested reactivation owing to recurrent symptoms. After delivery, SNM was not working in 40% of patients and required revision. The authors concluded that the decision regarding SNM activation or deactivation should be individualized and, due to the limited evidence, no clear advice could be given.

## 8. Programming Algorithm

The sacral neuromodulator operates through programming parameters, the adjustability of which is essential for therapeutic purposes. These encompass the electrode configuration (selection of the anode and cathode), the amplitude of the electrical pulses (mA or V), the pulse frequency (Hz) and the duration of each electrical pulse, known as pulse width (μs). The configuration of parameters for therapeutic stimulation is chosen based on a fundamental rule: selecting the configuration that produces the best sensory response with the lowest pulse amplitude [77]. One of the major advantages of sacral neuromodulation therapy is the possibility to conduct the procedure in two stages, the first being a test phase, and the second being the permanent implantation. Therefore, since the first stage serves as a test phase to assess the effectiveness of the device for the patient, there is the possibility to avoid implanting an unnecessary foreign body and save money on a permanent stimulator if no benefits are observed. Follow-up during the trial phase is essential to intervene with any necessary adjustments in settings if there are no symptom improvements or if adverse effects occur. Typically, the test period can be extended up to 4 weeks, allowing for up to two programming modifications if no benefits are observed [78]. During these weeks, the patient completes questionnaires and fills out voiding diaries to guide the therapeutic decision. If the improvement is at least 50% compared to the baseline, the treatment is considered successful and the permanent implantation is carried out. During the test phase, nerve stimulation can be carried out in two ways: either through a temporary helical wire monopolar electrode, which is removed after the test period, or through the “staged tined lead procedure”. The latter involves the use of a quadripolar lead designed for long-term therapy, which remains in place if the treatment demonstrates benefits. The four electrodes (also named poles) of the quadripolar lead work as contact points for the cathode, so several programs are available to stimulate sacral spinal nerves and these can be changed according to patient sensations. Currently, the InterStim system has the capability to deliver energy through two different technologies. The Verify, an external stimulator used during the test phase, operates using constant current, while the IPG, the permanent stimulator, allows for energy delivery at a constant voltage. Neither has been proven to be superior to the other, as they can provide the same amount of energy if impedance remains stable. The initial programming is typically performed either immediately after lead implantation, if the procedure is done under local anesthesia, or a few hours after the procedure in the case of general anesthesia. The key difference between the Verify and the IPG lies in the fact that during the test phase, the Verify cannot function as an anode, unlike the IPG after the permanent implantation. Therefore, with the IPG in place, it is easier to identify the electrode with the best sensory response (on the midline area including the vagina, anus or perineum, corresponding to sacral nerve course) by testing each electrode (0, 1, 2, 3) one at a time as a cathode against the IPG acting as the anode in a monopolar stimulation mode [77,79,80]. Typically, however, the selected electrode corresponds to the one identified in the test phase. If discomfort occurs during stimulation, it may be beneficial to reduce the stimulation field by bringing the anode closer to the cathode. For instance, if 3−/0+ is causing discomfort, trying 3−/2+ might be a worthwhile adjustment [81]. The amplitude of stimulation dictates the energy transmitted to the sacral spinal nerve and hence the axon depolarization [82]. An accurate lead placement, facilitated by the use of a curved stylet, enables stimulation at lower amplitudes, resulting in reduced energy consumption to achieve the therapeutic effect [83]. Clinical practice strongly suggests the preference for selecting subsensory stimulations as therapeutic ones. This recommendation is motivated by the potential for battery conservation, the reduction of adverse effects associated with excessive stimulation and the avoidance of chronic nerve damage or accommodation that could lead to long-term therapy inefficacy [84]. It has been demonstrated that pulse frequency can be a crucial parameter for therapeutic success [85]. Conventionally, a standard frequency of 10–14 Hz is chosen for basic programming [86]. Nevertheless, studies have shown that some patients experiencing a loss of effectiveness in SNM may report improved functional outcomes following an increase in frequency up to 31 Hz [85]. Regarding pulse width, 210 μs is recognized as the standard [79]. In theory, increasing the pulse width reduces the current or voltage required to stimulate neural tissue [87]. However, in practice, it is infrequent to deviate from the standard, as modern IPGs allow for finer adjustments than pulse width to achieve greater nerve stimulation leading to clinical outcomes [88,89]. There are conflicting opinions regarding the feasibility of entrusting the patient with the responsibility to modify therapeutic programs in the case of a decrease in the effectiveness of the device or the onset of adverse effects. Patient compliance is of fundamental importance, as not everyone is equally adept at managing SNM. Nevertheless, the patient should be educated not to permanently increase the amplitude of the stimulus if the benefits are still present, even if they no longer perceive the constant stimulation.

## 9. Conclusions

Sacral neuromodulation (SNM) represents an established therapeutic option for urological patients suffering from idiopathic overactive bladder (OAB) syndrome with or without incontinence and non-obstructive urinary retention (NOR). Recent improvements in the devices, including MRI compatibility and the introduction of long-lasting and rechargeable batteries, make this a valid and attractive approach for clinicians and for patients who are non-responders or are unwilling to consider conservative options or medical treatments for their conditions. Further applications are emerging in bladder pain syndrome (IC/BPS) and neurogenic lower urinary tract dysfunction (NLUTD). Due to the lack of RCTs, it remains unclear which neurological patients are most suitable for sacral neuromodulation, but the available evidence supports its use in stroke, Parkinson’s disease (PD), MS and incomplete spinal cord injury. 

## Figures and Tables

**Table 1:** Overview of sacral neuromodulation systems and main features reported by the manufacturers.

	Size	Type	Battery Life	Average Price
Interstim X^tm^	44 mm × 51 mm	Non-rechargeable	10–15 years	EUR 10.000
Interstim Micro™	17 mm × 47 mm	Rechargeable	>15 years	EUR 14.000
Interstim II™	44 mm × 51 mm	Non-rechargeable	5 years	EUR 7.5000
Axonics r-SNM F15	10 cc	Non-rechargeable	10–20 years	N.A.
Axonics r-SNM R20	5 cc	Rechargeable	>20 years	N.A.

## Data Availability

No new data were created.
